# Soft Texture of Atlantic Salmon Fillets Is Associated with Glycogen Accumulation

**DOI:** 10.1371/journal.pone.0085551

**Published:** 2014-01-09

**Authors:** Jacob S. Torgersen, Erling Olaf Koppang, Lars H. Stien, Achim Kohler, Mona E. Pedersen, Turid Mørkøre

**Affiliations:** 1 Nofima AS, Ås, Norway; 2 Institute of Basic Sciences and Aquatic Medicine, Norwegian School of Veterinary Science, Oslo, Norway; 3 Institute of Marine Research, Bergen, Norway; 4 Department of Mathematical Sciences and Technology, Norwegian University of Life Sciences, Ås, Norway; Universitat de Barcelona, Spain

## Abstract

Atlantic salmon (*Salmo salar* L.) with soft fillets are not suited for manufacturing high quality products. Therefore fillets with insufficient firmness are downgraded, leading to severe economic losses to the farming and processing industries. In the current study, morphological characteristics of salmon fillets ranging from soft to hard were analysed. Different microscopic techniques were applied, including novel methods in this field of research: morphometric image analysis, periodic acid Schiff staining, immunofluorescence microscopy, transmission electron microscopy and fourier transform infrared microscopy. The results showed that the myocytes of soft muscle had detached cells with mitochondrial dysfunctions, large glycogen aggregates and enlarged inter cellular areas, void of extracellular matrix proteins, including lower amounts of sulfated glycoproteins. Myofibre-myofibre detachment and disappearance of the endomysium in soft muscles coincided with deterioration of important connective tissue constituents such as Collagen type I (Col I), Perlecan and Aggrecan. In summary our investigations show for the first time an association between soft flesh of Atlantic salmon and massive intracellular glycogen accumulation coinciding with degenerated mitochondria, myocyte detachment and altered extracellular matrix protein distribution. The results are important for further understanding the etiology of soft salmon.

## Introduction

Texture quality is important for consumer acceptability of Atlantic salmon and insufficient firmness causes downgrading in the processing industry [1]. The issue of muscle texture variation is complex and affected by both ante- and post-mortem factors. The amount and composition of connective tissue and muscle fibre density are among inherent characteristics found to affect muscle texture [2]–[7]. Post-mortem softening during storage is related to connective tissue degradation, which decrease adhesion between myocytes and the endomysium [8]. Additionally, increased muscle softness post-mortem correlates with proteolytic degradation of extracellular matrix and cell membrane constituents [9], [10]. There is little available evidence on the importance of post-mortem degradation of specific proteins supporting muscle fibre strength, but Caballero et al. reported that muscle softening and myofibre-myofibre detachment of sea bream (*Sparus aurata*) is related to degradation of cytoskeletal proteins; for example rapid breakdown of dystrophin [11]. *In vivo*, transcriptome profiling of muscle atrophy in rainbow trout has identified transcriptional responses and pathways involved, including up regulation of genes involved in proteolysis, aerobic metabolism and decreased extracellular matrix collagens [12]. In line with these results, recent gene expression profiling of farmed salmon revealed that sufficient firmness of salmon muscle was largely dependent on an efficient aerobic metabolism and rapid removal of damaged proteins [13].

This work is part of a larger study determining the underlying mechanisms related to salmon muscle texture [13], [14]. Here we present comprehensive morphological characterization of salmon fillets with texture ranging from soft to very firm. To elucidate a possible link between texture and muscle morphology, a number of histological approaches were applied, including morphometrical analysis, FT-IR microscopy, transmission electron microscopy and immunohistochemical techniques.

## Materials and Methods

### Ethics Statement

Farmed Atlantic salmon (*Salmo salar* L.) with an average body weight of 3.5 kg were selected among a resource population obtained from the breeding company SalmoBreed AS, Norway. The fish were reared throughout their entire production cycle in a farming cage that is similar to commercial production units at Nofima research station (Averøy, Norway), which is approved by the Norwegian Animal Research Authority (NARA). The fish were treated as production fish up to sacrifice and sampling, and slaughtering was performed by the staff at Nofima Research station. Hence, no NARA approval was required according to Dr. G Baeverfjord (Nofima), appointed by NARA.

### Experimental Design

The fish (n = 944 individuals) were transferred to seawater in May 2007 as 1+ smolts. All fish were sacrificed in September 2008 by percussive stunning and bled in fresh seawater after cutting the left gill arches. The fish were filleted immediately after bleeding (pre-rigor) and muscle for histological examination was sampled from 120 fish. Thereafter the fillets were stored on ice for four days before instrumental determination of fillet firmness. Based on the mechanical texture analyses, 15 salmon with firmness ranging from very soft to hard were selected for muscle cell morphological analyses using haematoxylin and eosin (HE) staining, periodic acid Schiff (PAS) staining, and examination using immunofluorescence (IF). Three soft and three hard textured individuals were selected for transmission electron microscopy (TEM) and fourier transform infrared spectroscopy (FTIR) analyses. For further details on the fish material, experimental design, physiochemical properties and transcriptome profiling see Larsson et al. who used the same sample material [13].

### Texture Analysis

Instrumental determination of firmness was performed using a TA-XT2, Stable Micro Systems Ltd. (Surrey, England) by pressing a flat-ended cylinder (12.5 mm diameter, type P/0.5) into the epaxial fillet part, just anterior to the dorsal fin. The compression analyses were performed perpendicular to the muscle fibres at 1 mm/sec. The force required to puncture the fillet surface (breaking force, Newton) was registered from the resulting time-force graphs. The breaking force analysed in raw salmon fillets was shown to correlate significantly to sensory assessment of firmness of both raw and smoked salmon [15].

### Histological Preparation

Muscle biopsies were carefully sampled from the episkeletal muscle about 4 cm anterior to the dorsal fin. For paraffin embedding, the samples were fixed in 4% paraformaldehyde for 24 hours, whereas 2.5% glutaraldehyde was applied for samples to be examined with TEM. For FTIR analyses, histological staining and immunofluorescence paraffin was removed from the sections prior to rehydration in decreasing ethanol concentrations. Morphometric analysis of sections was carried out on HE stained material. Muscle glycogen was visualized using periodic acid Schiff (PAS) staining [16]. TEM samples were processed as previously described [17].

### Morphological Analysis for Muscle Cells

Microscopy images of HE stained muscle sections from each specimen were obtained using an Observer Z1 Zeiss microscope and then analysed using Matlab v7.2 (The MathWorks Inc., Natick, MA, USA). Briefly, semi-automatic segmentation scripts identified the borders of the cells in each image and calculated the cell area, number of cernels, eccentricity, convexity, cell to cell distance and pericellular area of a total of 200 cells from each specimen. The results on morphological characteristics were analysed using ANOVA (SAS Institute Inc, USA).

### FT-IR Measurement

An optical IR spotlight 400 microscope (Perkin Elmer) coupled to a Spectrum 400 FT-IR spectrometer (Perkin Elmer, UK) was used to measure the tissue sections. Spectra were collected from different connective tissue regions in the frequency range 4000 to 750 cm^−1^ using a mercury cadmium telluride (MCT) detector, and with spectral resolution of 8 cm^−1^, 64 scans per pixel and spectral interval of 4 cm^−1^. A background spectrum of the ZnSe substrate was recorded before each sample measurement in order to account for variation in water vapour and CO_2_ level. Second derivative of the spectra were taken applying the Savitzky-Golay algoritm before further preprocessing by extended multiplicative signal corrections (EMSC) in The Unscrambler version 9.2 (Camo Process AS, Oslo, Norway) to remove multiplicative and wavenumber independent and dependent baselines [18]. To analyze the main variation in FT-IR absorbance bands of connective tissue between firm and soft fish, data analysis was performed using principal component analysis (PCA) without standardization of variables.

### Immunofluorescence (IF)

Microwave facilitated IF was initiated by antigen retrieval for 20 min in 10 mM Tris-HCl pH 10.0. Permeabilization was carried out using 1% Triton in PBST for 20 min, before blocking in 2% dried milk diluted in PBST. Salmon specific Col I (Biologo, Germany), Perlecan (Chemicon, Germany) [19] and Aggrecan (Santa Cruz Biotechnology, USA) [19] primary antibodies were diluted in PBST and subjected to 3 min intermittent microwave incubation at 195 W [20]. The sections were washed thoroughly in PBST before incubation with Alexa conjugated secondary antibodies (Life Technologies Ltd, UK) as described above. Negative controls were incubated with secondary antibodies only. After successive washings in PBST, the slides were cover-slipped using Prolong Gold antifade (Life Technologies). Images were captured on a Zeiss Axio Observer Z1 equipped with the Apotome system for structured illumination and analysed using AxioVision software (Carl Zeiss Microimaging GmbH, Jena, Germany).

## Results

### Texture

The fillet firmness (breaking force, N) of the salmon used for muscle cell morphological analyses ranged from 6.6 N –20.9 N. Hence the whole range from soft to hard muscle was covered. The fish were divided into five groups according to the fillet firmness analyses (n = 3 within each group): soft (6.6–7.5 N), low firmness (8.6–9.5 N), medium firmness (9.7–12.5 N), high firmness (13.1–16.7 N) and hard (17.7–20.9 N).

### Histomorphometry

**Figure 1:**
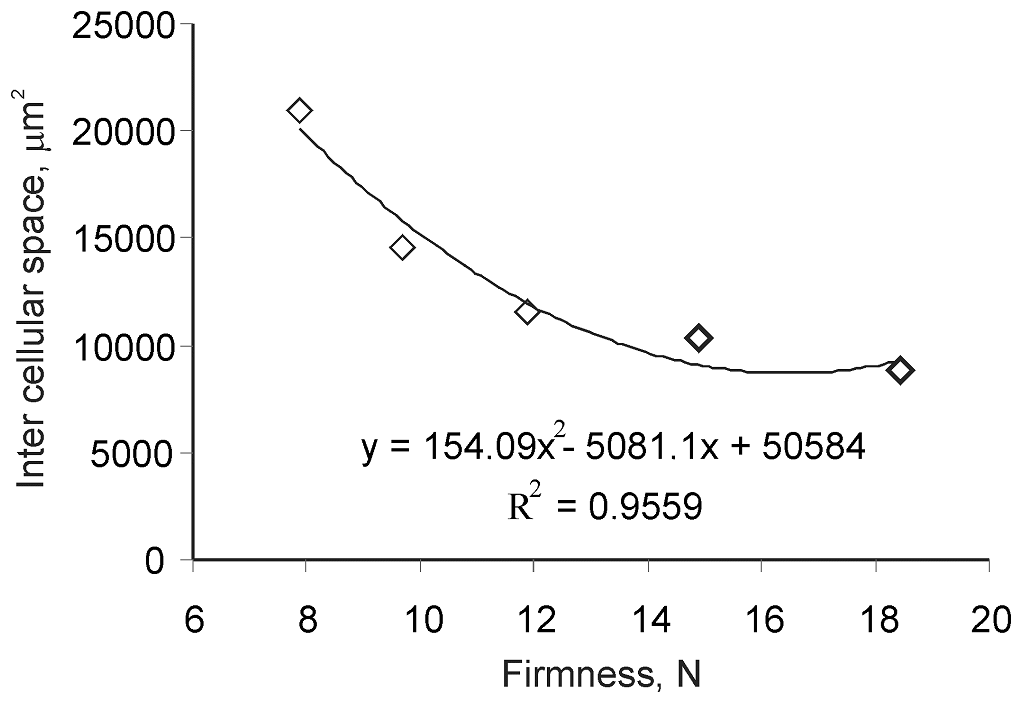
Regression analysis of histomorphometric data shows a highly significant relationship between intercellular space and soft muscle texture of farmed Atlantic salmon. Each data point represents the average of each texture group: soft, low firmness, medium firmness, high firmness and hard (n = 3 per group).

Image processing of histology cross sections of skeletal muscle revealed a curvilinear relationship between firmness and pericellular area (Fig. 1). Other morphometric phenotypes, including cell area, cell shape and the number of intracellular nuclei proved less accurate for discriminating between different textures.

### FT-IR

**Figure 2:**
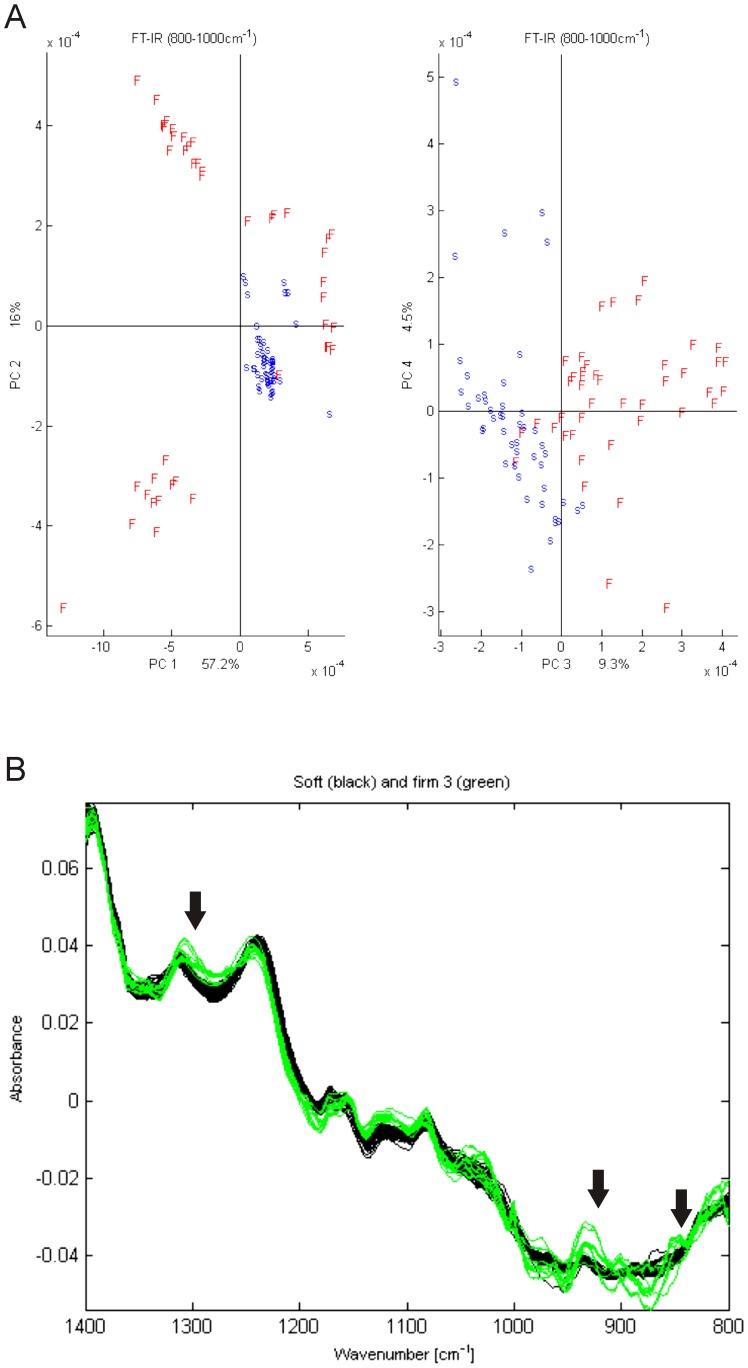
PCA score plots of connective tissue in hard (F) and soft (S) salmon fillets using the frequency bins in region of 800–1000 cm^−1^ as variables (A). Endomysial FT-IR absorbance spectra in hard and soft fish. A higher absorbance value was obtained at peak positions 850 cm^−1^, 925 cm^−1^ and 1314 cm^−1^ of firm salmon (green line) compared to soft salmon fillets (black line). These peak positions can be derived from sulfated GAGs of Aggrecan [21], and is consistent with a higher amount of Aggrecan or similar glycoproteins in this connective tissue region of firm fish (B).

FT-IR was used to determine sulfated glycosaminoglycans (GAGs) in connective tissue of hard and soft fish. Analyses of the endomysium were obtained in the junction between three or more myocytes. The results showed that hard muscle differed significantly from soft muscle within the spectral region of 800–1000 cm^−1^ (PCA score plot, Fig. 2A), which represents the typical area of sulfated glycosaminoglycans [21]. A higher absorbance value at peak positions 850 cm^−1^ band, 925 cm^−1^ and 1314 cm^−1^ of hard muscle compared to soft muscles was detected (Fig. 2B). Peak positions at 1314 cm^−1^ and between 800–1000 cm^−1^ have previously been described to correspond to Aggrecan carrying sulfated GAGs [21], [22].

### Ultrastructure Analysis and PAS Staining

**Figure 3:**
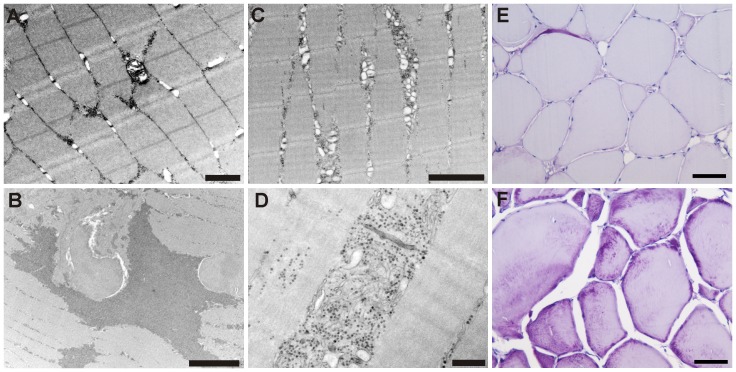
Ultra-thin section of muscle cell from individual with hard texture quality. There are some deposits of glycogen granules between the myofibrils. Uranyl acetate and lead citrate stain, bar = 1 µm (A). Ultra-thin section of muscle cell from individual with soft texture. Large accumulations of glycogen granules are seen as darker, irregular areas, and several myofibrils are degenerated. Uranyl acetate and lead citrate stain, bar = 5 µm (B). Ultra-thin section of muscle cell from individuals with soft texture. Glycogen granules may be seen within and between the myofibrils. Accumulations seem to be co-occurring with degenerated mitochondria. Uranyl acetate and lead citrate stain, bar = 400 nm (C). Ultra-thin section of muscle cell from individual affected with soft texture quality. Swollen mitochondria and accumulations of glycogen granules (black) are seen between the myofibrils. Uranyl acetate and lead citrate stain, bar = 2 µm (D). PAS stained section of muscle from individual with hard texture quality. The myocytes show very limited appearance of glycogen (purple color) and the muscle cells seem to be firmly attached to one another. bar = 50 µm. F) PAS stained section of muscle from individual with soft texture. Irregular and intense glycogen staining unevenly distributed within muscle cells, associated with the cell membrane and also as extracellular deposits. The cells appear disintegrated and detached from one another. bar = 50 µm (E).

Transmission electron microscopy of hard (Fig. 3A) and soft muscles revealed the occurrence of abundant granulated material with an appearance conformal with glycogen accumulation (Fig. 3B). Such granules were detected between myofibrils (Fig. 3C), frequently associated with swollen or even degenerated mitochondria (Fig. 3D), but also within myofibrils. Occasionally, degenerated myofibrils had been replaced by a substantial accumulation of glycogen (Fig. 3F). Fish with soft texture also displayed PAS stained material within muscle cells and in extracellular debris adjacent to the affected cells. Myocytes in such tissue seemed detached, displaying an open space devoid of any tissue structures between them. In comparison, fish with hard texture displayed very faint PAS staining and the muscle cells seemed to be firmly attached to one another (Fig. 3E). TEM investigations of such muscle showed normal-appearing mitochondria and sparse occurrence of glycogen granules.

### Immunofluorescence

**Figure 4:**
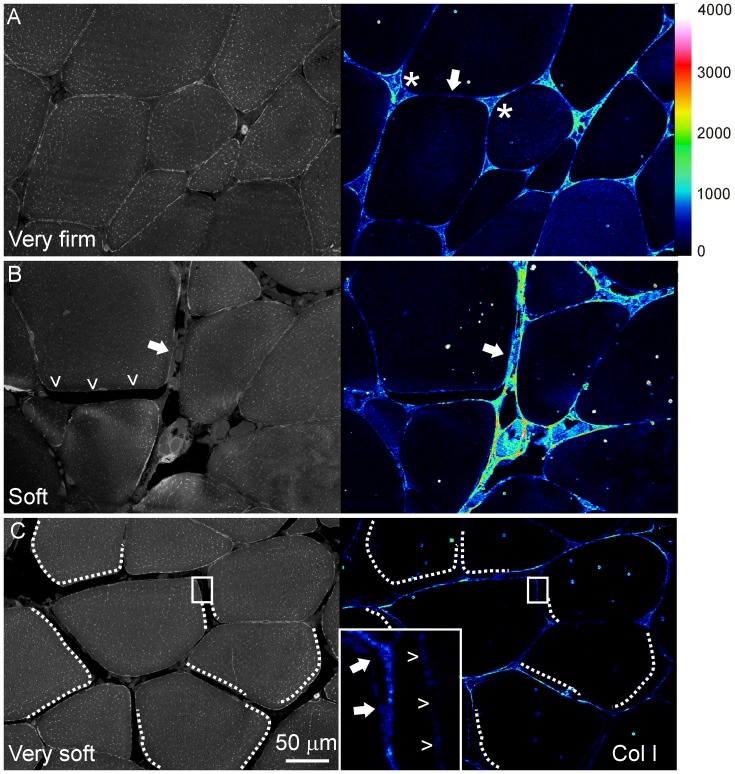
Muscle morphology shown as grey scale images of autofluorescence and immunofluorescence analysis of Col I as LUT images, respectively. The muscle analysed was sampled between two perimysial layers of the dorsal fillets. A) Hard muscles show a fine line of Col I between two adjacent cells (arrow) and more abundant content where more than two cells attach (asterisks). B) Col I rich fibrotic material (arrow) and total lack of endomysium (arrow head) in muscle with low firmness. C) Myocytes in a section of soft muscle appear detached and lack Col I along the stippled lines. Inset show a higher magnification with Col I along the sarcolemma of one myocyte (arrows), whereas the neighbouring cell have almost non-detectable fluorescence (arrow heads).

**Figure 5:**
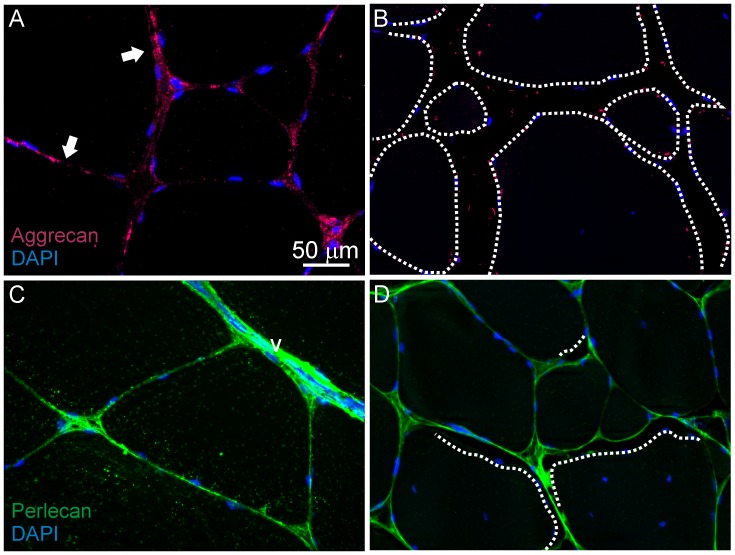
Immunofluorescence analysis of Aggrecan (A, B) and Perlecan (C, D) in hard and soft muscles. A) Aggrecan is apparent along the endomysium of hard muscles. B) In soft muscles, less protein is evident as well as aggregates. Stippled lines indicate the sarcolemma of detached myocytes. C) Perlecan in the endomysium of a hard muscle. Note the abundant staining in the blood vessel (v). D) In soft muscles Perlecan is lost in the sarcolemma of detached myocytes (stippled lines).

Analysis of immunofluorescence stained muscle sections was carried out to investigate changes in the extracellular matrix and cell membranes. In hard muscle, Col I was detected throughout the finely organized endomysium, with highest abundance in the junctions between three-four myocytes (Fig. 4A). Muscle with low and medium firmness showed increased Col I accumulation in the endomysium, which appeared fibrotic and wider compared with muscle with higher firmness (Fig. 4B). Col I staining of neighbouring detached myocytes in soft muscle was very weak or absent. Interestingly, only one of the affected cells featured loss of Col I, whereas the other affected myocytes had retained Col I fluorescence (Fig. 4C). Similar to Col I, Perlecan was present in the endomysium of hard muscles (Fig. 5A), whereas pericellular content was almost lost in myocytes detached from their neighbouring cells (Fig. 5B). Microscopy for Aggrecan in hard muscle showed similar spatial distribution as the two other proteins (Fig. 5C), though the soft muscles featured aggregates and loss of pericellular distribution (Fig. 5D).

## Discussion

Image segmentation is a powerful tool to accurately analyse cell morphology. In muscle tissue, this approach has successfully been applied to describe morphology and myopathic conditions [23], [24]. The histomorphometric image analysis in the present study showed significantly enlarged extracellular space in soft muscles, whereas no significant correlation was observed between texture and myofibre characteristics *per se*. These results coincide with Mørkøre et. al, who concluded that texture of conventionally farmed salmon is clearly multifactorial, where muscle fibre size is not a major determinant [25].

The combination of myofibre detachments, fibrosis, swollen or degraded mitochondria and glycogen granules in-between the myofibrils in the soft skeletal muscle suggest the possibility of an uncharacterized glycogen storage myopathy, similar to glycogen storage myopathies and mitochondrial myopathy in equine (For review see [26]) and humans [27]. Glycogen accumulation in the soft phenotype salmon can, however, be a symptom of the underlying cause rather than the cause itself, for example impaired glycogen metabolism as a consequence of mitochondrial dysfunction. It is well documented that biochemical changes play an important role for the texture of fish fillets. In particular rapid acidification post-mortem from anaerobic glycolysis and a low final pH have been have been associated with softness [28], [29], possibly due to reduced connective tissue strength [30], denaturation of proteins and increased proteolysis [30]. Expression profiles of mitochondrial genes of the same individuals as those analysed in the present study strongly suggest an association between soft flesh and higher levels of anaerobic metabolism [13]. Although fillet texture showed a significant genetic variation (heritability 0.16) [13], it is not possible to determine whether the metabolic or morphological properties of the skeletal muscle were inherited. Future studies should reveal the frequency and underlying causes to abnormal glycogen accumulation in salmon skeletal muscle in order to reduce the problem with soft texture and to avoid secondary pathology. In addition, advances in understanding underlying mechanisms are required to define potential treatments (e.g. through diet).

The association between fillet firmness and the amount and spatial distribution of Col I in the endomysium is in accordance with Bremner, who reported that degradation and distortion of collagen in the endomysium is of importance for texture of fish fillets [31]. Fibrosis in salmon muscle with less severe myopathy and abundance of Col I coincided with increased gene expression of *col I* [13]. Similar observations are valid for human muscular dystrophies, where Col I and III are accumulated in fibrotic tissue [32]. In a recent publication based on the same specimens as those in the present study, it was concluded that firmness was not related to the total amount of collagen [14]. Their results were based on larger muscle samples including both muscle segments (myosepta) and connective tissue sheets between the muscle segments (myocommata), hence the main part of the collagen naturally originated from the myocommatal connective tissue. In the present study, amounts and distribution of Col I were studied in myofibre membranes of the myosepta. Despite the low quantity, the results suggest that collagen of the myosepta may have a significant impact on the fillet texture. The gaping phenomena, however, is presumably more significantly dependent on the collagenous tissue of the myocommata, as gaping is associated with myofibre-myocommata detachments caused by disruption of collagen fibrils [8].

Perlecan is a heparan sulfate proteoglycan that locates to the cell membrane and extracellular matrix. Most studies have focused on its role in bony tissues, but during skeletal muscle development, Perlecan plays a crucial role and acts as an important regulator of several growth factor signalling pathways and lipid metabolism [33], [34]. Loss of Perlecan activity in mouse results in hypertrophy [35], thus the decreased amounts of Perlecan observed in this study may have implications for muscle morphology in salmon. The association between lipid metabolism and Perlecan is also interesting as fillet firmness of Atlantic salmon depends largely on metabolic properties of the skeletal muscle [13], where aerobic metabolism using lipids as fuel appear to play a major role for desired fillet texture. Aggrecan has been little studied regarding its role in skeletal muscle. Immunofluorescence of endomysium confirmed decreased amounts of Aggrecan and was supported by the FT-IR data indicating decreased amount of Aggrecan or similar glycoproteins in the soft muscles. Microscopy also confirmed the formation of aggregates and spatial changes. Soft textured salmon muscles are more prone to water release [36], and it may be that Aggrecan could play a role in this process, because of its water binding properties [37].

### Conclusion

We report for the first time an association between soft flesh of Atlantic salmon and massive intracellular glycogen accumulation coinciding with swollen and degenerated mitochondria, myocyte detachment and altered extracellular matrix protein distribution. The results are important for further understanding the etiology of soft salmon.
